# Dislocation of Total Hip Arthroplasty of Femoral Neck Fracture in the Elderly: A Narrative Review

**DOI:** 10.7759/cureus.46307

**Published:** 2023-10-01

**Authors:** Emmanouil Skotidis, Kyriakos Bekas, Ioannis Kechagias, Ioannis Tsakonas - Ntervakos, Spyridon P Galanakos, Konstantinos Kateros

**Affiliations:** 1 First Orthopaedic Department, G. Gennimatas General Hospital, Athens, GRC; 2 Orthopaedic Department, Primary Health Care Corporation, Athens, GRC

**Keywords:** fracture femoral neck, hemi arthroplasty, hip dislocation, surgical approach, total hip arthroplasty (tha)

## Abstract

Femoral neck fracture (FNF) is a common and devastating injury in the elderly population. The incidence of FNF is expected to increase in the future, particularly in the aging population. The displaced intracapsular FNF is replaced with a reconstruction prosthesis. These treatment options typically include hemiarthroplasty (HA) or total hip arthroplasty (THA). Dislocation after THA can be a significant complication, leading to increased hospital costs and patient dissatisfaction. This narrative review aims to investigate the potential risk factors for dislocation following THA after FNF.

A systematic literature search was conducted, and 21 studies met the inclusion criteria. The studies included a total of 1703 patients who underwent THA after FNF. The majority of the patients were women, and the average age of participants was 76.2 years. The studies were primarily conducted by the orthopedic and traumatology departments. The surgical approach used for THA varied, with the anterior approach being associated with lower dislocation rates compared to the posterior approach.

The analysis of surgical volume revealed that high-volume hospitals had lower dislocation rates compared to low-volume hospitals. Eight studies reported postoperative Harris Hip Scores (HHS), with higher HHS scores correlating with lower dislocation rates. Body mass index (BMI) was mentioned in 11 studies, and a normal BMI range was associated with lower dislocation rates compared to the overweight group.

Rehabilitation protocols, particularly early initiation of physiotherapy, showed promising results in reducing dislocation rates. Additionally, the type of prosthesis used in the acetabulum was found to influence dislocation rates, with dual mobility cups demonstrating lower rates compared to single cups.

In conclusion, several factors may contribute to the risk of dislocation following THA after FNF. These include the surgical approach, surgical volume, postoperative HHS scores, BMI, rehabilitation protocols, and the type of acetabular cup used. Further research is needed to better understand these risk factors and develop strategies to minimize dislocation rates and improve patient outcomes.

## Introduction and background

Femoral neck fractures (FNFs) can have devastating consequences, particularly in the elderly population [1-3]. Among the elderly who lead an active lifestyle, FNF is one of the most common fractures [4,5], with an annual incidence of approximately 1.7 million [6]. Projections indicate that the incidence of FNF will increase to approximately six million cases per year by 2050 [4,7]. In contrast to younger individuals who typically experience FNF due to high-energy trauma, simple falls and deficiencies in bone mineralization (e.g., estrogen imbalances, vitamin D deficiency, and secondary hyperparathyroidism) are the most common causes in the elderly population [8-10].

When it comes to treating FNF, there are several options available, including open reduction internal fixation, hemiarthroplasty (HA), and total hip arthroplasty (THA) [10-14]. In the elderly population, either HA or THA is considered the optimal treatment [2,15]. Recent studies suggest that THA yields better outcomes compared to HA for FNF [7,16,17]. Moreover, evidence has shown that elderly active individuals who live independently and undergo THA for FNF experience higher satisfaction and lower reoperation rates compared to those undergone hemi arthroplasty [17,18-20].

The most common complications following THA are dislocation, infection, and aseptic loosening. Dislocation after THA imposes an additional financial burden on hospitals [21,22-25]. The male-to-female ratio for dislocation after THA is approximately 1:3 [13,21]. Numerous studies have demonstrated that the dislocation rate after THA for FNF is significantly higher than that observed after elective THA [5,11,13,22]. Although estimating the precise dislocation rate following THA for FNF is challenging, recent evidence suggests a range between 2% and 7% [5,13,23].

Therefore, the aim of this study was to conduct a comprehensive narrative review of the existing literature and explore potential risk factors associated with dislocation following THA after FNF.

## Review

Search strategy

We conducted a narrative review of the literature using PubMed and Scopus, specifically targeting retrospective and randomized control trials with a focus on studies classified as level I and level II evidence. After an initial screening of 1886 research papers, we identified and included a total of 21 studies that met our inclusion criteria. We performed a narrative review of the literature in PubMed and Scopus.

A total of 1886 potentially relevant studies were first identified during the database search, of which 1684 were initially removed because they dealt with irrelevant aspects of FNF. The remaining 202 studies were reviewed based on the title/abstract, leading to the exclusion of 139 studies with irrelevant titles or abstracts. Out of the 63 articles that were selected for reading, only 21 met the inclusion criteria of this systemic review.

The inclusion criteria were as follows: patients aged ≥65 years with a primary diagnosis of FNF who underwent THA, regardless of whether it was cemented or uncemented; patients with FNFs unrelated to any medical condition that could be the cause of FNF; studies conducted from the year 2000 onward, including retrospective studies and controlled trial studies; English-language studies conducted in any country or region; and publications with a primary author.

The exclusion criteria were as follows: unavailability, lack of direct relevance to FNF, investigation of HA, and unclear description of randomized methods for the treatment of irrelevant FNFs with THA. Meta-analyses, systematic reviews, case studies, case reports, and studies with insufficient, incomplete, or inappropriate data were also excluded.

**Figure 1:**
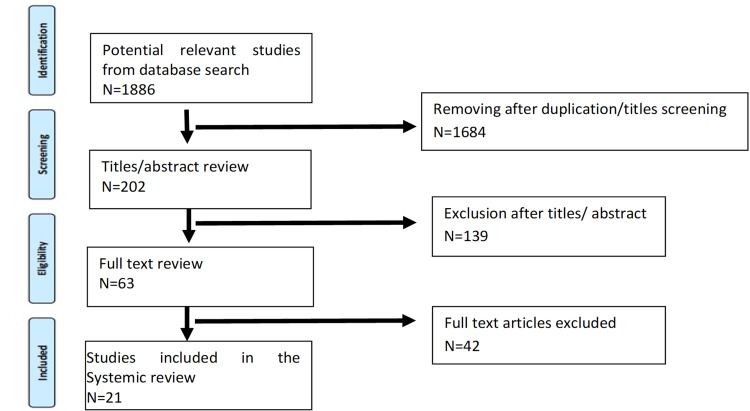
Method used to collect all the data using the PRISMA flow chart PRISMA: Preferred Reporting Items for Systematic Reviews and Meta-Analyses.

We used the Preferred Reporting Items for Systematic Reviews and Meta-Analyses (PRISMA) flow chart to collect all the data as shown in Figure 1.

Conducting and documenting

A detailed search log was meticulously maintained to track all relevant research data. This log included information such as the time period and timeframe used for searching, the keywords utilized, the databases searched, and the search engines employed. The literature research was conducted independently by two authors, both of whom read the abstracts and excluded articles that were unrelated to the topic of interest. The databases searched included MEDLINE, ScienceDirect, and Cochrane Review, specifically focusing on articles related to THA dislocation after FNF. Additionally, a manual search was performed through the bibliography of each selected article to identify any relevant studies. Supplementary strategies, such as exploring cited articles and manually searching references and citations, were employed to locate potentially suitable articles. The keywords used in the search process were as follows: "femoral neck fracture and total hip arthroplasty," "femoral neck fracture and arthroplasty," and "femoral neck fracture and hip dislocation after total hip arthroplasty."

Data analysis

**Figure 2:**
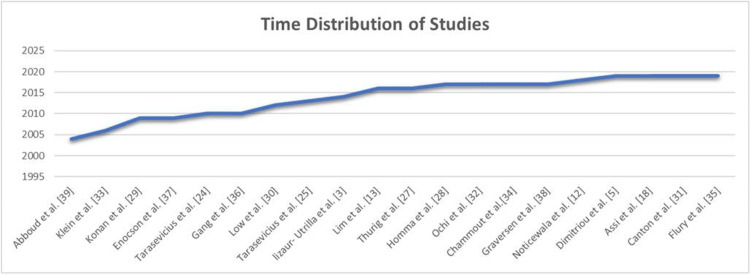
Time distribution of studies from 2004 to 2019 Source: Refs. [3,5,12,13,18,24,25,27-39].

The 21 studies comprised 1703 examined patients (482 male and 1221 female) with dislocation following THA after FNF [24-39]. Most of the patients who underwent THA after FNF were women (n = 1221). In this systemic review, the male-to-female ratio was 1:4 similar to the primary THA ratio of 1:3 [28,37,38]. The average age of the participants was 76.2 years. We found Abboud et al. [39] to be the first to investigate the dislocation after THA following FNF. The overall data distribution showed that the last decade had the most studies focusing on dislocation after THA following FNF. Interestingly, most of the studies were conducted between 2016 and 2019 (Figure 2).

Dislocation based on different departments

Eleven of the studies relating to dislocation following THA after FNF were conducted by orthopedic and traumatology departments (OTDs), eight studies were conducted by orthopedic departments (ODs), and very few were conducted by trauma departments (TDs) [26-40]. The OTD studies included 1100 patients (318 male and 782 female) (Table 1), with an overall dislocation rate of 4.6%. The OD studies included 475 patients (124 male and 351 female), with an overall dislocation rate of 2.1%. The TD studies [31,38] included only 128 patients (40 male and 88 female), with an overall dislocation rate of 2.3%.

**Table 1:** Dislocation rates based on different departments

Departments	n	Dislocation	Age (mean years)
Orthopedics [12,18,24,25,27,28,34,39]	475 (M:124/W351)	10 (2.1%)	76.2
Trauma and orthopedics [3,5,13,26,29,30,32,33,35–37]	1100 (M:318/W:782)	51 (4.6%)	76.1
Trauma centers [31,38]	128 (M:40/ W:88)	3 (2.3%)	75.7

Surgical approach

In this systemic review, the anterior surgical approach was used in the highest number of subjects and had the lowest rate of dislocation (Table 2). The posterior approach was the second most common surgical technique, but it had the highest rate of dislocation (5.2%). Our results showed that the posterior approach more than doubled the chances for dislocation compared to the anterior approach.

**Table 2:** Dislocation rates based on different surgical approaches

Surgical approach	No	Dislocation
Posterior [12,13,18,24,25,31,32,35,37]	712	37 (5.2%)
Anterior [16,18,24,28–30,36,37]	818	17 (2.1%)
Lateral	305	10 (3.3%)

Based on the surgical approach data, the anterior approach was mainly used in OTDs and TDs. In contrast, the posterior approach was mainly used in ODs. The anterior approach had the lowest rates of dislocation in all departments, whereas the posterior approach appeared to have the highest rates of dislocation (Table 3).

**Table 3:** Dislocation rates based on different surgical approaches and department levels

Departments	Surgical Approach		
	Anterior	Posterior	Lateral
Orthopedics (No/Dislocation) [12,18,24,25]	109/0	256/7 (2.7%)	110/3 (2.7%)
Trauma and orthopedics (No/Dislocation) [5,13,30,41]	581/15 (2.6%)	414/29 (7%)	195/7 (3.6%)
Trauma center (No/Dislocation) [31,38]	108/2 (1.9%)	20/1 (5%)	0/0

Surgical volume: surgeons

Seventeen studies referred to hospital volumes. High-volume hospitals had a dislocation rate of 3.4%, whereas low-volume hospitals had a dislocation rate of 5.8% (Table 4). The collected data for surgical volume were from different countries with different volumes for THA.

**Table 4:** Dislocation rates based on department’s volume

Department's volume	No/Dislocation
High volume [3,5,12,26-29,34-39]	977/33 (3.4%)
Low volume [13,31-33]	463/27 (5.8%)

Harris Hip Score (HHS) and dislocation rates

In all the studies, only 11 used postoperative HHS. It had been replied by 811 patients (266 male and 545 female), with an average score of 88.5 in five studies, which included 298 patients with a “good” HHS (Table 5); this group reported a dislocation rate of 3%. In contrast, six studies scored “excellent,” consisting of 513 patients. This group reported a dislocation rate of 1.5% dislocations [40-43].

**Table 5:** HHS and dislocation rates HHS: Harris Hip Scores.

HHS	Score (mean)	n	Dislocation
Good [2,23,26,32,35]	88.5	298	9 (3%)
Excellent [14,18,28,29,36,38]	92.8	513	8 (1.5%)

BMI and dislocation

Among the 11 studies that mentioned body mass index (BMI), the average BMI was 23.9 (n = 859). Those individuals within the normal BMI range had a lower dislocation rate, which can be compared to the dislocation rate reported for the overweight group (Table 6).

**Table 6:** BMI and dislocation rates

BMI	n	Dislocation
Normal [5-24,42]	392	9
Overweight [9,25-29]	467	20

Rehabilitation

Rehabilitation was mentioned in nine studies; the overall number of subjects was 607, with only 2.3% reporting dislocation, which is a relatively low rate of dislocation compared to those mentioned previously in our study (2%-7%). Most of the studies mentioned that the physiotherapy protocol began on the first postoperative day, with the only exception being the study from Wang et al. [44] wherein the physiotherapy protocol began on day 3.

THA prosthesis

Acetabular Cup

Comparing the data based on the type of prosthesis used in the acetabulum, the studies using a dual mobility cup (DMC) had lower dislocation rates compared to those using a single mobility cup (SMC) as shown in Table 7.

**Table 7:** Correlation between the type of acetabular cup and dislocation rates

Department	Dual mobility cup (DMC) [18,26,27,33,35–37,39]			Single cup (SC) [3,5,12,13,24,25,27–31,34,36,38,45]		
	Anterior [24,37]	Posterior [18,35–37]	Lateral [31,38]	Anterior [5,12,24,25,27-31,38]	Posterior [13,35,36,45]	Lateral [3,33,34]
Orthopedics (No/Dislocation)	73/0	125/0	0/0	36/0	138/7	115/3
Trauma and orthopedics (No/Dislocation)	0/0	166/3	50/0	581/15	249/26	143/7
Trauma center (No/Dislocation)	0/0	0/0	0/0	108/2	20/1	0/0

More specifically, cases with a DMC and the anterior approach [28,32] had no cases of dislocation, but those with a DMC and the posterior approach [18,35-37] had a dislocation rate of 1.8%. The dislocation rate of the posterior approach combined with an SC was 10.4% [13,35,36,40], which was almost six times higher compared to the same group using a DMC [35-37]. Moreover, using either the posterior approach and an SC or the lateral approach and an SC [28,34] halved the risk of dislocation compared to the combinations of the posterior approach and SC [13,32,35,36] and lateral approach and SC [3,29].

Femoral Stem

Concerning the type of femoral prosthesis used, 1170 THA cases used cemented femoral stems [7,15,16,21,30,34-38,40-42], and 530 THA cases used cementless femoral stems [3,12,24-29,32,34,38,39]. In cemented stems [5,12,13,18,29-33,35-37], the risk of dislocation was twice as high compared to cementless stems [3,12,24-29,32,34,38,39] (Table 8). There were no reports of dislocation in those with a DMC in combination with a cementless stem [28,31,32] or a cementless cup [33,35,36].

**Table 8:** Dislocation rates compared between the surgical approach and the use of cement

Surgical approach	Femoral stem		Acetabular cup	
	Cemented [7,15,16,21,30,34-38,40-42]	Cementless [3,12,24–29,32,34,38,39]	Cemented [3,30,34]	Cementless [29,30,34]
Anterior (No/Dislocation) [5,13,27,28,32,35-37]	621/14 (2.2%)	197/2 (1%)	42/0	525/12 (2.2%)
Posterior (No/Dislocation) [12,13,18,24,25,29,30,36,37]	377/32 (8.4%)	79/2 (2.5%)	187/11 (5.9%)	151/4 (2.6%)
Lateral (No/Dislocation) [3,31,33,34]	55/1 (1.8%)	254/9 (3.5%)	85/1 (1.2%)	221/6 (2.7%)

Further analysis of the data from an anterior approach in combination with cementless stem and DMC [27,39] reveals the lowest dislocation rate of 1% (Table 9).

**Table 9:** Dislocation rates compared between the surgical approach and the use of cement with dual mobility cup

Dual mobility cup (DMC)	Femoral stem		Acetabular cup	
	Cemented [18,24]	Cementless [2,28]	Cemented [18,24]	Cementless [2,28]
Anterior approach (No/Dislocation) [28]	0/0	73/0	0/0	73/0
Posterior approach (No/Dislocation) [18,24]	291/3	0/0	83/0	208/3 (1.4%)
Lateral approach (No/Dislocation) [2]	20/0	60/0	19/0	61/0

On the contrary, the posterior approach with SC and cemented stem [12,13,25,31,32,36] gives the highest rate of dislocation of 9.5% (Table 10).

**Table 10:** Dislocation rates compared between the surgical approach and the use of cement with a single cup

Single cup (SC)	Femoral stem		Acetabular cup	
	Cemented [7,15,16,21,30,34-38,40-42]	Cementless [3,12,24–29,32,34,38,39]	Cemented [3,30,34]	Cementless [29,30,34]
Anterior (No/Dislocation) [5,13,27,32,35-37]	621/15 (2.4%)	154/2 (1.3%)	42/0	482/12 (2.5%)
Posterior (No/Dislocation) [12,13,25,29,30,36,37]	335/32 (9.5%)	79/2 (2.5%)	187/11 (5.9%)	151/4 (2.6%)
Lateral (No/Dislocation) [3,33,34]	55/1 (1.8%)	224/9 (2.4%)	66/4 (6%)	193/6 (3.1%)

Considering center type, ODs [12,24,25,27,28,34,39] and TDs [27] using cementless stems had the lowest dislocation rates of 1.6% and 2.3%, respectively (Table 11).

**Table 11:** Correlation between femoral stem and dislocation

Departments	Femoral stem	
	Cemented [3,27,29,30,34]	Cementless [5,12,29,30,31,36]
Orthopedics (No/Dislocation) [12,29,30]	170/5	301/5
Trauma and orthopedics (No/Dislocation) [3,5,31,34]	958/45	143/6
Trauma centers (No/Dislocation) [27,36]	42/1	86/2

Hospitalization

Only nine studies mentioned the number of days until hospital discharge, with an overall average of eight days. The posterior approach had the highest number of days until discharge at 9.7 days, whereas the lateral approach had the fewest number of days until discharge (Table 12).

**Table 12:** Surgical approaches and hospitalization

Surgical approach	Hospitalization (mean days)
Anterior [16,18,28,29,36]	8.2
Posterior [16,29,35]	9.7
Lateral [23,31,32]	6.4

The posterior approach using either a DMC (Table 13) or a SC (Table 14) had the highest number of days until discharge compared to the other two surgical approaches.

**Table 13:** Dual mobility cap and hospitalization DMC: Dual mobility cup.

DMC	Hospitalization (mean days)
Anterior approach [3,5,30,38]	No information
Posterior approach [13,31,37]	11
Lateral approach [26,33,34]	7.3

**Table 14:** Single mobility acetabular cup and hospitalization SC: Single cup.

SC	Hospitalization (mean days)
Anterior approach [4,7,16,35,36]	8.2
Posterior approach [16,36]	9.1
Lateral approach [39]	4.7

Lastly, patients who stay more days in the hospital have a higher chance of dislocation (Table 15).

**Table 15:** Hospitalization and dislocation rate

Hospitalization	Dislocation Rates
>7 days (No/Dislocation) [7,36,38,39]	142/4
<7 days (No/Dislocation) [16,31,35,36,42,43]	376/13

Discussion

The aim of this article is to systematically review the current literature available to evaluate the dislocation rate after THA following FNF. Regarding the current literature, there are numerous possible risk factors for dislocation. The data from this systematic review reveal a significant gender disparity in patients undergoing THA following FNF, with a male-to-female ratio of approximately 1:4. Interestingly, this ratio is consistent with the primary THA male-to-female ratio (1:3) reported in the literature [45]. The predominance of female patients in the studies may be due to various factors, such as a higher incidence of FNF in elderly women, differences in bone density and morphology between genders, and variations in healthcare-seeking behaviors. Risk factors for dislocation can be divided into two main categories: surgical factors and patient factors.

The surgical factors include various factors such as surgeon experience, type of center, surgical approach (anterior/posterior/lateral), and type of prosthesis (stem, cup) [6,29,40,41]. Our results showed that the anterior approach had the lowest dislocation rate compared to the posterior and lateral approaches. This finding is consistent with previous research highlighting the advantages of the anterior approach in terms of reduced soft tissue disruption and improved joint stability, leading to lower dislocation rates [7,16,32,33,37,40-42]. Similarly, a recent multisystemic study by Cebatorius et al. [46] suggested that the anterior approach had a lower dislocation rate compared to the posterior approach. On the other hand, the posterior approach showed the highest dislocation rate, with the risk of dislocation being twice as high as that of the anterior approach [15,16,21,29,31,34,35,41,42]. Surgeons should consider these findings when selecting the surgical approach for THA following FNF while considering patient-specific factors and the risk-benefit profile of each approach. Common complications of the anterior approach include intraoperative fractures and transient nerve palsy [43-45].

Our results showed that high-volume hospitals had a lower dislocation rate compared to low-volume hospitals [3,7,15,29-33,36-39,44], which emphasizes the importance of surgical experience and case volume in achieving better surgical outcomes, including lower dislocation rates. High-volume centers are likely to have more experienced surgeons, specialized care teams, and standardized protocols, contributing to improved patient outcomes. Surgeons should consider referring patients to high-volume centers for THA following FNF to potentially reduce the risk of dislocation and other postoperative complications.

Regarding the type of prosthesis used, our results showed that DMCs in the acetabulum were associated with lower dislocation rates compared to SCs [29,36,37]. The dislocation rate with DMCs ranged from 0% to 1.1% [41-43]. The use of cementless stems also appeared to have lower dislocation rates compared to cemented stems [3,15,32,33,35-39,44]. Lizaur-Utrilla et al. [3] argued that cementless stems give better results in osteoporotic patients. Hybrid prostheses (i.e., uncemented cups and cemented stems) are also commonly used and show relatively good results [44,45]. Additionally, the combination of a DMC with a cementless stem and the anterior approach showed the lowest dislocation rate [33,37]. These findings suggest that the choice of implant design can significantly impact dislocation rates. Surgeons should carefully consider the type of prosthesis, especially in high-risk patients, and consider DMCs in cases where stability is a primary concern. There is no widespread agreement on the most suitable prosthesis type.

Patient risk factors for dislocation included modified and unmodified risk factors. The unmodified risk factors included age, American Society of Anesthesiologists score, and muscular incoordination [6,29]. Neuromuscular diseases can contribute to instability after THA, leading to higher dislocation rates (5%-8%) [2,7,45]. The modified risk factors included BMI and HHS. Studies have shown a correlation between complications after THA and BMI [7]. The results of this study indicated that patients with a normal BMI had a lower dislocation rate compared to those with overweight (2.1% vs. 4.2%, respectively) [3,7,15,16,31,33,37-40]. This finding is in line with previous studies that have shown an association between higher BMI and increased risk of dislocation after THA [40]. Overweight patients may have altered joint mechanics and increased soft tissue laxity, making them more susceptible to dislocation. Therefore, surgeons should take BMI into consideration when assessing the risk of dislocation in patients undergoing THA following FNF and provide appropriate preoperative counseling and postoperative care for overweight individuals.

Our results also showed that early physiotherapy and rehabilitation protocols, usually starting from the first postoperative day, are associated with a lower dislocation rate [3,7,31,32,36,40,41,43,44]. Early mobilization and strengthening of the hip joint are crucial in promoting stability and reducing the risk of dislocation. Additionally, patients with longer hospital stays (>7 days) had a higher chance of dislocation compared to those with shorter stays [3,7,31,32,36,40,41,43,44]. Prolonged hospitalization may result in muscle weakness and joint stiffness, potentially increasing the risk of postoperative dislocation. Therefore, surgeons and healthcare providers should implement structured and early rehabilitation programs and consider optimizing hospitalization times to improve patient outcomes.

Based on the current findings, surgeons should consider the anterior approach when performing THA following FNF as it appears to have the lowest dislocation rates. Furthermore, the use of DMCs in combination with cementless stems shows promise in reducing dislocation rates, particularly in high-risk patients. Surgeons should also emphasize early rehabilitation and physiotherapy protocols to promote joint stability and optimize patient outcomes. Collaborative efforts between orthopedic and TDs, along with increased surgical volumes, may lead to improved patient outcomes and lower dislocation rates.

This systematic review provides valuable insights into the factors influencing dislocation rates in THA following FNF. Understanding the impact of surgical approach, prosthesis type, patient characteristics, and postoperative care is crucial in reducing the risk of dislocation and improving overall patient outcomes. Implementing evidence-based practices and tailored treatment strategies can pave the way for better surgical outcomes and enhanced patient satisfaction in THA patients following FNF.

Our review has certain limitations. First, numerous postoperative (deep vein thrombosis, pulmonary embolism, infection) and intraoperative complications (such as periprosthetic fractures, instability, bleeding, and iatrogenic nerve damage) were assessed by only a few studies, which decreased the grade of evidence. Another limitation of randomized controlled trials (RCTs) are relatively small sample size and limited follow-up (one to five years). As a result, the ability to draw long-term conclusions and assess the durability of interventions is hampered. Moreover, the subanalysis of specific fracture configurations is restricted in its scope. To address these limitations and provide more conclusive insights, there is a clear need for additional high-quality randomized studies that can better elucidate the advantages and outcomes associated with THA after FNF.

## Conclusions

The study included 1703 patients who underwent THA after FNF, with a mean age of 76.2 years. The findings indicated that the parameters, such as anterior approach, high surgical volume hospitals, good HHS, normal BMI, early rehabilitation, and the use of DMCs, may contribute to lower dislocation rates following THA after FNF. However, it is important to note that these findings are based on the selected studies, and further research is needed to validate these results.
