# Correction: Dislocation of Total Hip Arthroplasty of Femoral Neck Fracture in the Elderly: A Narrative Review

**DOI:** 10.7759/cureus.c264

**Published:** 2025-08-26

**Authors:** Emmanouil Skotidis, Kyriakos Bekas, Ioannis Kechagias, Ioannis Tsakonas - Ntervakos, Spyridon P Galanakos, Konstantinos Kateros

**Affiliations:** 1 First Orthopaedic Department, G. Gennimatas General Hospital, Athens, GRC; 2 Orthopaedic Department, Primary Health Care Corporation, Athens, GRC

This article has been corrected to fix the following typo present in the PRISMA diagram and the first paragraph of the Review section:

"Studies included in the systematic review" has been corrected from 20 to 21.

